# Supplementary figures and images for: The critical importance of mask seals on respirator performance: An analytical and simulation approach
Source: PLoS One. 2021 Feb 17;16(2):e0246720. doi: 10.1371/journal.pone.0246720 (PMC7888670; doi:10.1371/journal.pone.0246720)

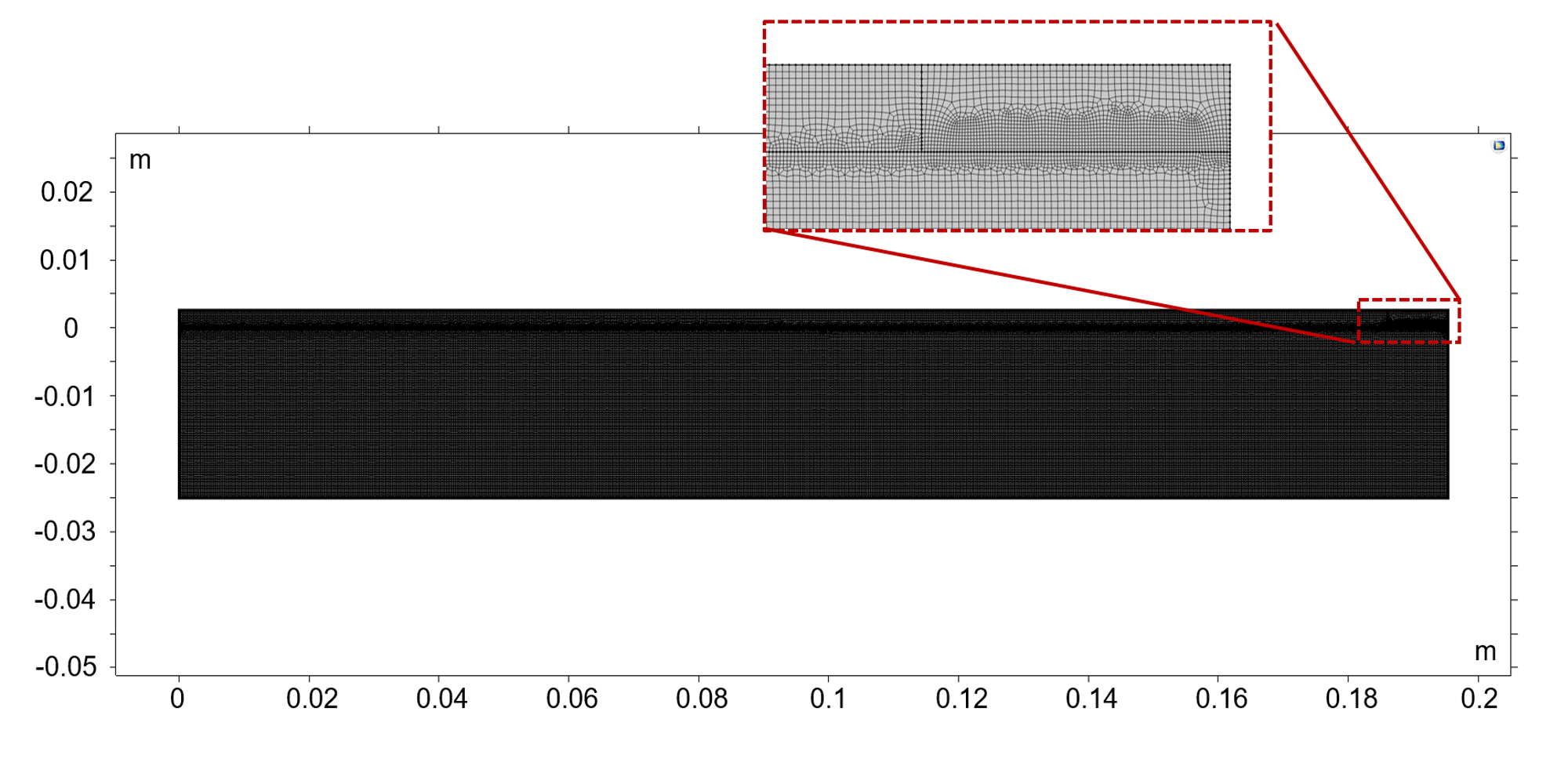

Supplement: S1 Fig — (TIF) [file pone.0246720.s001.tif]

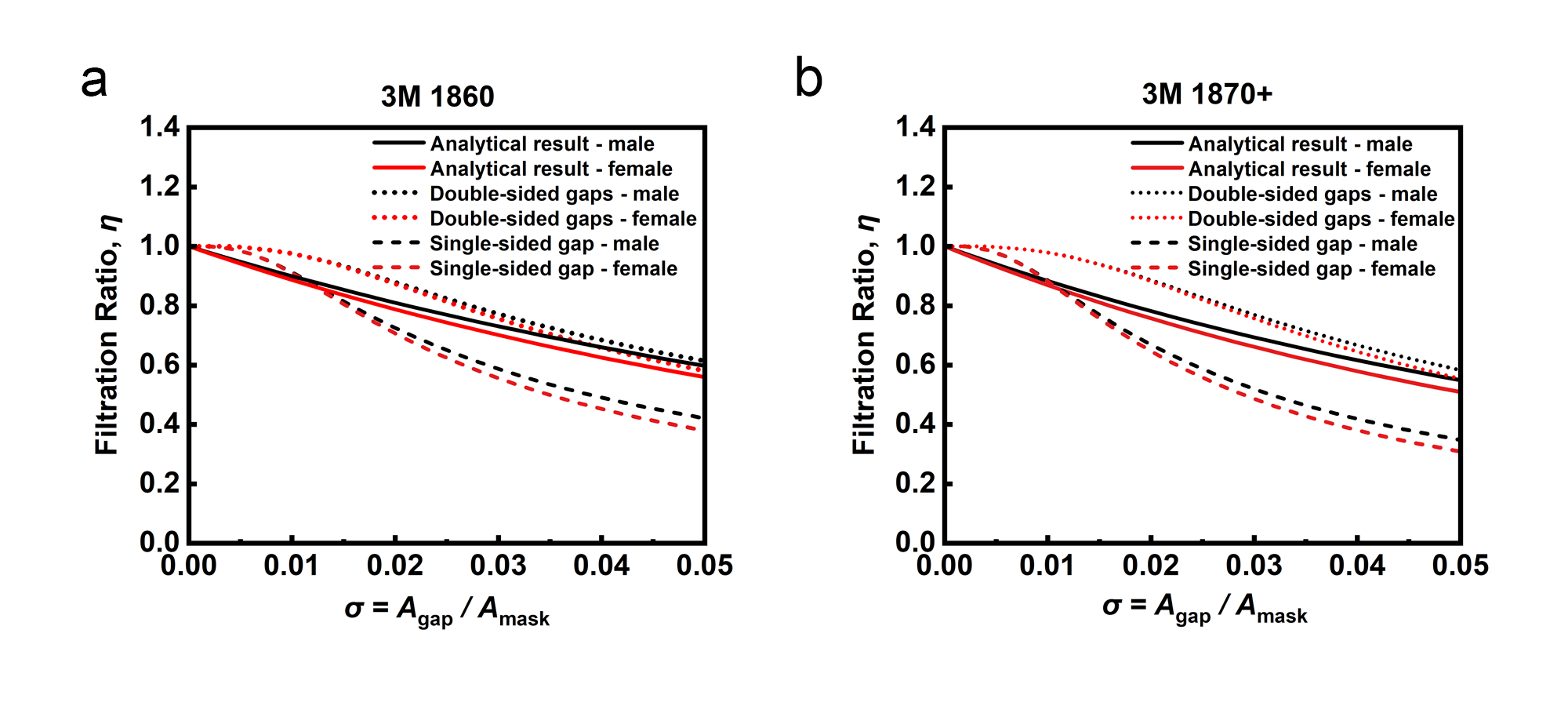

Supplement: S2 Fig — For (a) 3M 1860 and (b) 3M 1870+ masks. (TIF) [file pone.0246720.s002.tif]

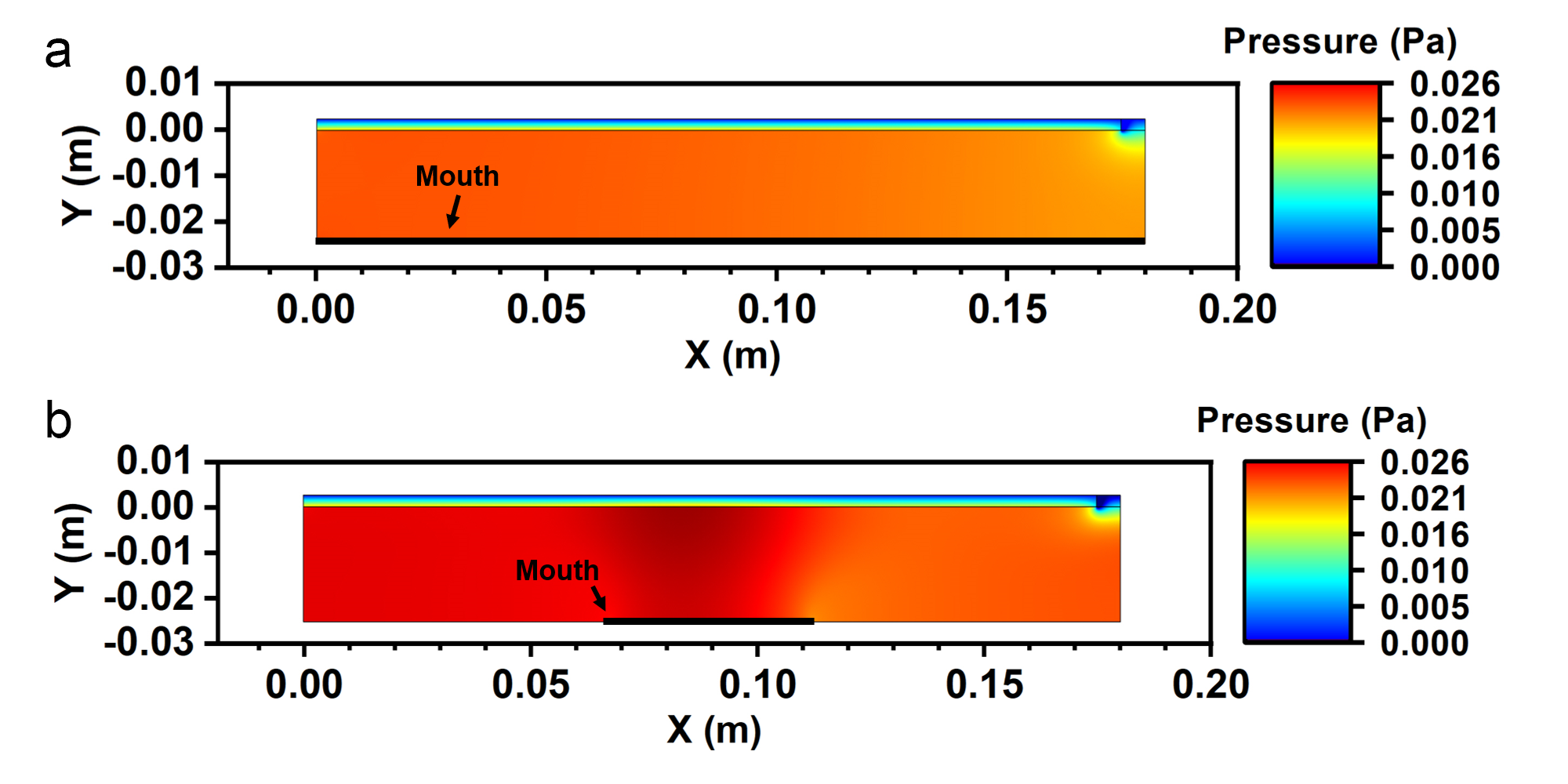

Supplement: S3 Fig — For (a) Wmouth = Wmask + Wgap and (b) Wmouth = 0.0463 m. With σ = Wmask/Wgap = 0.03 and Qtot = 0.00035 m3·s-1. (TIF) [file pone.0246720.s003.tif]
